# Comparative-Effectiveness of Oral Beta-Lactams and Fluoroquinolones for Stepdown Therapy in Patients with Enterobacterales Bloodstream Infections: A Retrospective Cohort Study

**DOI:** 10.7150/ijms.80621

**Published:** 2023-02-13

**Authors:** Lauren Bjork, Teri Hopkins, Linda Yang, Chengwen Teng, Xavier Jones, Jose Cadena, Elizabeth Walter, Christopher R. Frei

**Affiliations:** 1 South Texas Veterans Health Care System, San Antonio, TX, USA; 2 Long School of Medicine, University of Texas Health San Antonio, San Antonio, TX, USA; 3 College of Pharmacy, The University of Texas at Austin, Austin, TX, USA; 4 College of Pharmacy, The University of South Carolina, Columbia, SC, USA

**Keywords:** beta-lactams, fluoroquinolones, bloodstream infections

## Abstract

**Background:** This study compares treatment failure for patients who received oral beta-lactams (BLs) and fluoroquinolones (FQs) for stepdown treatment of Enterobacterales bloodstream infections (BSIs).

**Methods:** We conducted a single-center, retrospective, age- and sex-matched, cohort study, at a Veterans Affairs (VA) hospital in South Texas. Eligible patients were at least 18 years of age with a monomicrobial BSI treated with a single oral BL or FQ antibiotic. Treatment failure was defined as recurrence or all-cause mortality within 90 days of documented BSI. Bivariate (chi-square, Fisher's Exact, and Wilcoxon Rank Sum) and multivariate (logistic regression) statistical tests were used to compare groups.

**Results:** A total of 130 patients were included in this study, with 65 patients per group. Groups were well balanced with respect to exact age, sex assigned at birth, Caucasian race, source control, intensive care unit admission, and Charlson Comorbidity Index. Importantly, 60% of patients in the BL group had cultures that were resistant to FQs and 71% were prescribed cefpodoxime. Patients in the BL group had higher median (interquartile range [IQR]) Pitt bacteremia scores than those in the FQ group: 2 (1-4) vs. 1 (1-2), p=0.04. Patients in the BL group also had a higher median (IQR) duration of intravenous (IV) antibiotics than those in the FQ group: 5 (3-7) vs. 4 (3-5), p=0.02. Treatment failure was statistically comparable for patients in the BL and FQ groups: 15% vs. 12%, p=0.61. This finding was consistent in a multivariate logistic regression model with group (BL vs. FQ) as the independent variable, treatment failure as the dependent variable, and Pitt bacteremia score and duration of IV antibiotics as covariates (OR: 0.76, 95% CI: 0.27-2.18). One patient in the FQ group experienced *Clostridioides difficile* infection.

**Conclusion:** This study suggests that BLs may be as effective as FQs for oral stepdown treatment of Enterobacterales BSI without the potential associated risks. Furthermore, in the setting of FQ-resistant Enterobacterales BSI secondary to urinary source, third generation oral cephalosporins (i.e., cefpodoxime) may be reasonable alternatives.

## Background

Gram-negative bloodstream infections (BSI) are commonly treated with an initial course of intravenous (IV) antibiotics followed by definitive oral stepdown therapy in clinically stable patients with adequate source control 1-3. Historically, oral antibiotics with high bioavailability (i.e., fluoroquinolones [FQ]) have been utilized as definitive therapy 4-10. However, FQ are associated with concerning adverse drug events (ADEs) as well as the emergence of drug resistance 11,12. Safe and effective alternative oral agents are needed for definitive treatment of Enterobacterales BSI 13. Prior to this study, there was limited available data to support the use of oral beta-lactams (BLs) for the definitive treatment of BSI 14-16. The primary concern is that BLs have low oral bioavailability compared to FQs and might require longer durations of therapy. Therefore, patients may have an increased risk of treatment failure with BSIs 14. In reviewing the existing clinical literature, it is crucial to account for total duration of therapy 17-23.

One retrospective cohort study by Kutob and colleagues demonstrated the effectiveness of oral antibiotics with high bioavailability for definitive therapy of gram-negative BSI. Additionally, they found that the risk of treatment failure increases as bioavailability of the oral regimen declines 14. Another retrospective cohort study by Mercuro and colleagues also sought to determine if treatment failure rates were comparable between BLs and FQs 15. Their findings suggest that oral beta-lactams were non-inferior to fluoroquinolones as stepdown therapy for Enterobacterales BSI, with fewer ADE 15. In a recent multicenter cohort study of patients with *E. coli, Klebsiella* spp., or *Proteus* spp. bacteremia from urine source, Sutton and colleagues found no significantly higher risk of recurrent bacteremia, though the overall risk was small (1.5% with BL antibiotics vs. 0.4% with FQ) 16.

Based on the available literature, there are an increasing number of studies assessing the effectiveness of BL therapy for the definitive oral treatment of Enterobacterales BSI 14-16,14-28. Providers may be hesitant to prescribe oral BLs for this indication, but often have no other oral options based on susceptibilities due to increasing FQ resistance. There is also heterogeneity amongst prior studies in terms of the antibiotics included, both with BL selection and the comparator, as some studies include both FQs and trimethoprim/sulfamethoxazole, and others only include FQs. Most of these studies do not report a significant number of patients that received cefpodoxime, and the probability of pharmacokinetic (PK) target attainment for cefpodoxime is unknown. This study aims to determine if treatment failure rates were comparable in patients who received a BL (primarily cefpodoxime) or FQ for oral stepdown treatment for Enterobacterales BSIs.

## Methods

### Study subjects and definitions

This was a single center retrospective chart review at the South Texas Veterans Health Care System in San Antonio, Texas from January 2008 - December 2018 of patients with BSI due to *Escherichia coli*, *Klebsiella* spp., or *Proteus* spp. and a single definitive oral antibiotic therapy. Data were collected from the Veterans Affairs electronic health record. Patients with an inpatient or outpatient pharmacy record for receipt of an oral BL or FQ within 30 days of documented BSI and a positive blood culture for *E. coli*, *Proteus* spp., or *Klebsiella* spp. were assessed for inclusion and exclusion criteria. Patients were included if they were at least 18 years of age with a monomicrobial BSI treated with a single definitive oral antibiotic. Patients treated with a total antibiotic duration < 6 days or > 21 days were excluded. Patients with FQ-resistant organisms were excluded in the FQ group and included in the BL group. In the BL group, none of the organisms appeared to be extended-spectrum-β-lactamase (ESBL) producing strains based on susceptibility patterns. Cefpodoxime susceptibility was assessed using disk diffusion, and all other susceptibilities were obtained with the automated testing platform, Vitek 2 (bioMérieux). There were fewer patients available for the BL group; therefore, groups were matched using a 1:1 ratio based on exact age and sex assigned at birth from the BL group. No patients from the BL group were lost because of matching. The primary outcome was treatment failure defined as a composite of microbiological recurrence at the primary site of infection or all-cause mortality within 90 days. Microbiological recurrence was defined as the isolation of any organism from the same site regardless of symptoms.

### Data collection and statistical analysis

Data included demographic characteristics, Charlson comorbidity index, Pitt Bacteremia score, infecting organism, type of inpatient IV antibiotic and duration, and definitive oral antibiotic with dose, frequency, and duration. Late stepdown therapy was defined as more than three days of IV antibiotics. Baseline demographics and clinical variables were compared between groups. Categorical data were analyzed using chi-square and Fisher's exact tests, while numerical data were analyzed using the Wilcoxon Rank Sum test. For treatment failure, a multivariate logistic regression model was constructed with group (BL vs. FQ) as the independent variable, treatment failure as the dependent variable, and Pitt bacteremia score and duration of IV antibiotics as covariates. Statistical significance was defined as p-values less than an alpha of 0.05. This study was approved by the University of Texas Health Science Center at San Antonio Institutional Review Board (IRB).

## Results

A total of 130 patients were included in this study, with sixty-five patients per group. Groups were well balanced with respect to median age, sex assigned at birth, Caucasian race, source control, intensive care unit admission, and Charlson Comorbidity Index (Table 1). Patients in the BL group had significantly higher median (interquartile range [IQR]) Pitt bacteremia scores than those in the FQ group: 2 (1-4) vs. 1 (1-2), p=0.04. Though not statistically significant, patients in the BL group had more urinary tract infections (UTIs), fewer intra-abdominal infections (IAIs), fewer *K. pneumoniae* infections, and more *P. mirabilis* infections. Importantly, 60% of patients in the BL group had FQ-resistant pathogens, which suggests that drug resistance was a factor in the selection of the oral stepdown antibiotic.

Groups were well balanced with respect to IV antibiotics, duration of oral therapy, and total duration of therapy (Table 2). Patients in the BL group had a significantly higher median (IQR) duration of IV antibiotics than those in the FQ group: 5 (3-7) vs. 4 (3-5), p=0.02 (Table 2). Though not statistically significant, patients in the BL group were more likely to have late (> 3 days) stepdown therapy. Patients in the BL group were most frequently stepped down to cefpodoxime (71%), amoxicillin/clavulanate (14%), cephalexin (12%), and amoxicillin (3%). Patients in the FQ group were most frequently stepped down to ciprofloxacin (85%) and levofloxacin (15%).

Treatment failure was statistically comparable for patients in the BL and FQ groups: 15% vs. 12%, p=0.61. This finding was consistent in a multivariate logistic regression model with group (BL vs. FQ) as the independent variable, treatment failure as the dependent variable, and Pitt bacteremia score and duration of IV antibiotics as covariates (OR: 0.76, 95% CI: 0.27-2.18). 90-day recurrence (BL: 9%, FQ: 5%, p=0.49) and 90-day all-cause mortality (BL: 6%, FQ 8%, p=1.00) were statistically comparable. One patient in the FQ group experienced *Clostridioides difficile* infection.

## Discussion

This retrospective study found comparable rates of treatment failure with oral BLs compared to FQs for definitive treatment of Enterobacterales BSI—primarily due to urine source. During the time period since this study was completed, a growing body of evidence to support oral BL treatment for this indication has been published 14-16,25-28. Our study is unique because it primarily compares the use of cefpodoxime vs. ciprofloxacin and reports the rates of FQ resistance (60%) within the BL group. Additionally, our study includes a 90-day follow-up period for the primary outcome, which differs from most studies conducted both prior to and after our study.

A prior 2016 retrospective cohort study suggests there is an inverse relationship between bioavailability of definitive oral antibiotics and risk of treatment failure in patients with gram-negative BSI, although only one patient in this study received cefpodoxime 14. In 2018, a retrospective observational cohort study was the first to compare BLs to FQs for oral stepdown therapy for Enterobacterales BSI. BLs were found to be non-inferior to FQs for the primary outcome of clinical success; microbiological success and 30-day readmissions were comparable between groups. Notably, patients were more likely to complete their BL therapy without experiencing ADEs compared to those treated with FQ therapy 15. In contrast, a recent 2022 retrospective matched cohort study found that patients receiving less bioavailable antibiotics compared to highly bioavailable antibiotics for gram negative BSI were associated with worse clinical outcomes 27. Recurrent BSI at 90 days was the primary factor driving the composite outcome. No data was provided on rates of FQ resistance in these two studies 14,15,27. Most BL patients were treated with either amoxicillin/clavulanate, amoxicillin, or cephalexin; cefpodoxime was not included.

Given practical challenges in microbiology labs, such as lack of updated susceptibility testing panels to reflect current CLSI breakpoints, some hospitals may need to rely on cefpodoxime over other more highly bioavailable oral BLs. For example, during the time period of this study, our lab was unable to routinely report cefazolin susceptibilities for this reason, and our automated susceptibility testing panel also did not include amoxicillin/clavulanic acid. Therefore, use of cefpodoxime was high despite agents like cephalexin and amoxicillin/clavulanic acid having more optimal serum concentrations and pharmacodynamic target attainment 14.

More recently, a large Veteran cohort study by Sutton and colleagues found no difference in risk of recurrent bacteremia between FQs and BLs for bacteremia with suspected urine source. Interestingly, though there was no statistically significant difference, recurrent bacteremia was three times more likely in the BL group (1.5% vs 0.4%). The authors concluded that even if this result had met statistical significance, in the setting of such infrequent events, this is not a clinically meaningful difference. Recurrence rates in our cohort were higher, which may be due to the longer 90-day follow-up period 16. None of the prior literature focused primarily on BSI due to urinary source. Saad and colleagues conducted a multicenter retrospective cohort study comparing clinical cure in patients with urinary tract and BSI who were stepped down to oral BLs compared to FQs. Of the 207 patients included, results suggest comparable clinical cure rates between groups at 30 days and none of the patients received cefpodoxime like other studies 28.

Robust PK studies have not been performed for cefpodoxime; therefore, a consensus guidance for treatment of gram-negative BSI recommends against routine use of cefpodoxime due to lack of data on PK target attainment 29. This study is one of the first to describe cefpodoxime use in the setting of Enterobacterales BSI. It could be a reasonable alternative to ciprofloxacin, especially in patients with adequate source control and urinary source of infection. Mogle and colleagues investigate the clinical considerations of prescribing BLs as step-down therapy for Enterobacterales BSI. Their primary concern is the uncertainty of BL dosing required to attain specific pharmacodynamic targets, especially in the setting of unknown minimum inhibitory concentrations (MICs) 24. At our institution, we are unable to obtain MICs for cefpodoxime, as susceptibility is reported based on Kirby-Bauer testing. The majority of cefpodoxime patients in our study (70%) received 200 mg by mouth (PO) twice daily (BID). To overcome the low oral bioavailability of cefpodoxime, higher doses (i.e., 400 mg PO BID) could be utilized as there are no major concerns for ADEs. In a recent meta-analysis of patients with Enterobacterales BSI, FQs may reduce the chances of recurrence compared to BLs. However, the authors noted that this may be attributed to the suboptimal dosing of BL regimens 25. Although lower cefpodoxime dosing was used in our study, outcomes appear to be comparable to those patients who received more highly bioavailable agents. Further clinical investigation is warranted to confirm if the high dose cefpodoxime regimen provides additional benefit over traditional dosing and to determine the adequate treatment duration.

In our cohort, total duration of therapy was approximately 14 days. A recent randomized controlled trial demonstrated that 7 days was non-inferior to 14 days for uncomplicated gram-negative bacteremia 23. However, oral BLs were underrepresented in this study (< 20% of overall cohort), which limits the ability to apply these findings when oral BLs are used as stepdown therapy. A desirability of outcome ranking (DOOR) analysis including this study suggests that short course (7 days) of antibiotics has comparable clinical outcomes to conventional course (14 days) in this patient population 30. Given the limited data for shorter durations of therapy when oral BLs are used, the 14-day duration of therapy used in our study may still be clinically applicable. In addition of total treatment duration, the average duration of IV therapy was comparable to other studies at approximately 5 days 17-23,28,30. It is important to note that IV duration in our study was on average one day shorter in the FQ group (4 days vs. 5 days). The clinical significance of this difference in IV duration is unknown.

Strengths of this study were that the groups were matched based on exact age and sex assigned at birth. Despite the retrospective design, groups were well balanced overall regarding baseline characteristics. The two areas where the groups differed most were the source of infection (more IAI in the FQ group) and infecting pathogen (more *K. pneumoniae* in the FQ group and more *P. mirabilis* in the BL group). Secondly, source control was obtained in most patients. Third, these data add to our limited knowledge in utilizing oral third generation cephalosporins in this patient population, which are known to achieve lower serum concentrations than more commonly used oral BLs. Lastly, rates of treatment failure were consistent with prior publications 14,15.

Limitations include the retrospective design, small sample size, and inadequate study power. We did a sample size calculation before the study, but did not find enough patients in the BL group to meet the sample size requirements. We screened and included all patients in the BL group (n=65) that met study criteria, plus an equal number of matched patients from the FQ group (n=65), so this was a population-based study versus a sample-based study for the BL group. The effect sizes were very small (< 5%) for all comparisons; these effect sizes were below the limit of detection for this study; therefore, it is possible to have type II error (failure to detect a statistically significant difference when one exists).

We did not know the source of infection at the time of group assignment. We determined the source of infection later, during the chart review. Also, with so few patients available for the BL group, we did not want to impose too many matching criteria as it would drop our BL group size even more. We could not include source of infection in the final multivariate model, because so many different sources made the model unstable. We recommend that if someone were to repeat this study in a larger health system, they should limit the study to only patients with urinary tract infections, because that was the source of infection for 80% of the patients in our BL group.

To account for potential confounders and any selection bias, Pitt Bacteremia Scores, Charlson Comorbidity Indexes, and ICU admission were collected and reported. These were found to be comparable between groups, although Pitt Bacteremia Score was higher in the BL group. This could potentially put the BL patients at higher risk of mortality. In addition to source and severity of illness, there was potential for changes in prescribing practices over the 10-year period studied. This is unlikely to impact our results, as the two groups were well balanced by date of positive blood culture. Another limitation of the retrospective design was the inability to closely monitor and identify ADEs. The safety endpoint of *C. difficile* only occurred in one patient. Since the VA is a closed system, patients would have returned to the VA for further management and follow-up, although it is possible some ADEs were not captured. Additionally, since microbiological recurrence was defined as the isolation of any organism from the same site regardless of symptoms, we could have included asymptomatic patients. Lastly, this study included primarily elderly Caucasian males and may not be generalizable to other patient populations.

## Conclusion

Additional prospective research is needed to determine the optimal oral BL stepdown agents, including both dosing and treatment duration. This study suggests that BLs may be as effective as FQs for oral stepdown treatment of Enterobacterales BSI without the potential associated risks.

## Figures and Tables

**Table 1:** Baseline Characteristics

Characteristic	Beta-lactam Group (n = 65)	Fluoroquinolone Group (n = 65)	P-value
Age - median (IQR), years	66 (60-78)	66 (60-78)	1.00
Male sex (assigned at birth) - no. (%)	61 (94)	61 (94)	1.00
Caucasian - no. (%)	50 (77)	48 (74)	0.68
Source control - no. (%)	57 (88)	54 (83)	0.46
Intensive care unit (ICU) admission - no. (%)	16 (25)	10 (15)	0.19
Charlson Comorbidity Index - median (IQR)	6 (4-8) *	5 (3-8) ^%^	0.48
Pitt bacteremia score - median (IQR)	2 (1-4)	1 (1-2)	***0.04***
Source of infection - no. (%)			
Urinary tract (UTI)	52 (80)	45 (69)	0.16
Intra-abdominal (IAI)	6 (9)	14 (22)	0.05
Skin and soft tissue (SSTI)	5 (8)	5 (8)	1.00
Central line associated (CLABSI)	1 (2)	0 (0)	1.00
Pneumonia	0 (0)	1 (2)	1.00
Unknown	1 (2)	0 (0)	1.00
Blood culture pathogen - no. (%)			
*E. coli*	45 (69)	41 (63)	0.46
* K. pneumoniae*	11 (17)	20 (31)	0.06
* P. mirabilis*	9 (14)	4 (6)	0.14
Fluoroquinolone resistance - no. (%)	39 (60)	-	N/A

no. = number; IQR = interquartile range; SD = standard deviation * Includes 62/65 patients ^%^ Includes 64/65 patients

**Table 2:** Antibiotic Therapy

Treatment	Beta-lactam Group (n = 65)	Fluoroquinolone Group (n = 65)	P-value
Intravenous (IV) antibiotic - no. (%)			
Beta-lactam	62 (95)	61 (94)	1.00
Fluoroquinolone	2 (3)	4 (6)	0.68
Aminoglycoside	1 (2)	0 (0)	1.00
Duration of IV therapy - median (IQR), days	5 (3-7)	4 (3-5)	***0.02***
Oral (PO) antibiotic - no. (%)			
Beta-lactams			
Cefpodoxime	46 (71)	--	N/A
Amoxicillin/clavulanate	9 (14)	--	N/A
Cephalexin	8 (12)	--	N/A
Amoxicillin	2 (3)	--	N/A
Fluoroquinolones			
Ciprofloxacin	--	55 (85)	N/A
Levofloxacin	--	10 (15)	N/A
Duration of PO therapy - median (IQR), days	10 (7-11)	10 (7-12)	0.32
Stepdown therapy (IV to PO)			
Early (≤ 3 days) - no. (%)	22 (34)	32 (49)	0.08
Late (> 3 days) - no. (%)	43 (66)	33 (51)	0.08
Total duration of therapy - median (IQR), days	14 (13-16)	14 (13-16)	0.41

no. = number; IQR = interquartile range; SD = standard deviation

## Abbreviations

ADE: adverse drug event
BID: twice daily
BL: beta-lactam
BSI: bloodstream infection
CI: confidence interval
FQ: fluoroquinolone
IAI: intraabdominal infection
ICU: intensive care unit
IV: intravenous
MIC: minimum inhibitory concentration
PO: by mouth
RR: risk ratio
UT: University of Texas
UTI: urinary tract infection
VA: Veterans Affairs

## References

[1] Barlam TF, Cosgrove SE, Abbo LM, MacDougall C, Schuetz AN, Septimus EJ, Srinivasan A, Dellit TH, Falck-Ytter YT, Fishman NO, Hamilton CW, Jenkins TC, Lipsett PA, Malani PN, May LS, Moran GJ, Neuhauser MM, Newland JG, Ohl CA, Samore MH, Seo SK, Trivedi KK (2016). Implementing an antibiotic stewardship program: Guidelines by the Infectious Diseases Society of America and the Society for Healthcare Epidemiology of America. Clin Infect Dis.

[2] Mermel LA, Allon M, Bouza E, Craven DE, Flynn P, O'Grady NP, Raad II, Rijnders BJ, Sherertz RJ, Warren DK (2009). Clinical practice guidelines for the diagnosis and management of intravascular catheter-related infection: 2009 Update by the Infectious Diseases Society of America. Clin Infect Dis.

[3] Hooton TM, Bradley SF, Cardenas DD, Colgan R, Geerlings SE, Rice JC, Saint S, Schaeffer AJ, Tambayh PA, Tenke P, Nicolle LE, Infectious Diseases Society of A (2010). Diagnosis, prevention, and treatment of catheter-associated urinary tract infection in adults: 2009 International Clinical Practice Guidelines from the Infectious Diseases Society of America. Clin Infect Dis.

[4] Fass RJ, Plouffe JF, Russell JA (1989). Intravenous/oral ciprofloxacin versus ceftazidime in the treatment of serious infections. Am J Med.

[5] Peacock JE Jr, Pegram PS, Weber SF, Leone PA (1989). Prospective, randomized comparison of sequential intravenous followed by oral ciprofloxacin with intravenous ceftazidime in the treatment of serious infections. Am J Med.

[6] Paladino JA, Sperry HE, Backes JM, Gelber JA, Serrianne DJ, Cumbo TJ, Schentag JJ (1991). Clinical and economic evaluation of oral ciprofloxacin after an abbreviated course of intravenous antibiotics. Am J Med.

[7] Amodio-Groton M, Madu A, Madu CN, Briceland LL, Seligman M, McMaster P, Miller MH (1996). Sequential parenteral and oral ciprofloxacin regimen versus parenteral therapy for bacteremia: a pharmacoeconomic analysis. Ann Pharmacother.

[8] Cunha BA (2006). Oral antibiotic therapy of serious systemic infections. Med Clin North Am.

[9] Park TY, Choi JS, Song TJ, Do JH, Choi SH, Oh HC (2014). Early oral antibiotic switch compared with conventional intravenous antibiotic therapy for acute cholangitis with bacteremia. Dig Dis Sci.

[10] Watson CM, Al-Hasan MN (2014). Bloodstream infections and central line-associated bloodstream infections. Surg Clin North Am.

[11] Shehab N, Lovegrove MC, Geller AI, Rose KO, Weidle NJ, Budnitz DS (2016). US emergency department visits for outpatient adverse drug events, 2013-2014. JAMA.

[12] Pepin J, Saheb N, Coulombe MA, Alary ME, Corriveau MP, Authier S, Leblanc M, Rivard G, Bettez M, Primeau V, Nguyen M, Jacob CE, Lanthier L (2005). Emergence of fluoroquinolones as the predominant risk factor for Clostridium difficile-associated diarrhea: a cohort study during an epidemic in Quebec. Clin Infect Dis.

[13] Brigmon MM, Bookstaver PB, Kohn J, Albrecht H, Al-Hasan MN (2015). Impact of fluoroquinolone resistance in Gram-negative bloodstream infections on healthcare utilization. Clin Microbiol Infect.

[14] Kutob LF, Justo JA, Bookstaver PB, Kohn J, Albrecht H, Al-Hasan MN (2016). Effectiveness of oral antibiotics for definitive therapy of Gram-negative bloodstream infections. Int J Antimicrob Agents.

[15] Mercuro NJ, Stogsdill P, Wungwattana M (2018). Retrospective analysis comparing oral stepdown therapy for enterobacteriaceae bloodstream infections: fluoroquinolones versus beta-lactams. Int J Antimicrob Agents.

[16] Sutton JD, Stevens VW, Chang NN, Khader K, Timbrook TT, Spivak ES (2020). Oral beta-Lactam antibiotics vs fluoroquinolones or trimethoprim-sulfamethoxazole for definitive treatment of Enterobacterales bacteremia from a urine source. JAMA Netw Open.

[17] Havey TC, Fowler RA, Daneman N (2011). Duration of antibiotic therapy for bacteremia: a systematic review and meta-analysis. Crit Care.

[18] Havey TC, Fowler RA, Pinto R, Elligsen M, Daneman N (2013). Duration of antibiotic therapy for critically ill patients with bloodstream infections: A retrospective cohort study. Can J Infect Dis Med Microbiol.

[19] Coats J, Rae N, Nathwani D (2013). What is the evidence for the duration of antibiotic therapy in Gram-negative bacteraemia caused by urinary tract infection? A systematic review of the literature. J Glob Antimicrob Resist.

[20] Swamy S, Sharma R (2016). Duration of treatment of Gram-negative bacteremia. Are shorter courses of antimicrobial therapy feasible?. Infectious Diseases in Clinical Practice.

[21] Nelson AN, Justo JA, Bookstaver PB, Kohn J, Albrecht H, Al-Hasan MN (2017). Optimal duration of antimicrobial therapy for uncomplicated Gram-negative bloodstream infections. Infection.

[22] Chotiprasitsakul D, Han JH, Cosgrove SE, Harris AD, Lautenbach E, Conley AT, Tolomeo P, Wise J, Tamma PD, Antibacterial Resistance Leadership G (2018). Comparing the outcomes of adults with Enterobacteriaceae bacteremia receiving short-course versus prolonged-course antibiotic therapy in a multicenter, propensity score-matched cohort. Clin Infect Dis.

[23] Yahav D, Franceschini E, Koppel F, Turjeman A, Babich T, Bitterman R, Neuberger A, Ghanem-Zoubi N, Santoro A, Eliakim-Raz N, Pertzov B, Steinmetz T, Stern A, Dickstein Y, Maroun E, Zayyad H, Bishara J, Alon D, Edel Y, Goldberg E, Venturelli C, Mussini C, Leibovici L, Paul M, Bacteremia Duration Study G (2019). Seven versus 14 days of antibiotic therapy for uncomplicated Gram-negative bacteremia: A noninferiority randomized controlled trial. Clin Infect Dis.

[24] Mogle BT, Beccari MV, Steele JM, Fazili T, Kufel WD (2019). Clinical considerations for oral beta-lactams as step-down therapy for Enterobacteriaceae bloodstream infections. Expert Opin Pharmacother.

[25] Punjabi C, Tien V, Meng L, Deresinski S, Holubar M (2019). Oral fluoroquinolone or trimethoprim-sulfamethoxazole vs. beta-lactams as step-down therapy for Enterobacteriaceae bacteremia: Systematic review and meta-analysis. Open Forum Infect Dis.

[26] Tamma PD, Conley AT, Cosgrove SE, Harris AD, Lautenbach E, Amoah J, Avdic E, Tolomeo P, Wise J, Subudhi S, Han JH, Antibacterial Resistance Leadership G (2019). Association of 30-day mortality with oral step-down vs continued intravenous therapy in patients hospitalized with Enterobacteriaceae bacteremia. JAMA Intern Med.

[27] Mponponsuo K, Brown KA, Fridman DJ, Johnstone J, Langford BJ, Lee SM, MacFadden DR, Patel SN, Schwartz KL, Daneman N (2022). Highly versus less bioavailable oral antibiotics in the treatment of gram negative bloodstream infections: a propensity-matched cohort analysis. Clin Microbiol Infect.

[28] Saad S, Mina N, Lee C, Afra K (2020). Oral beta-lactam step down in bacteremic E. coli urinary tract infections. BMC Infect Dis.

[29] Heil EL, Bork JT, Abbo LM, Barlam TF, Cosgrove SE, Davis A, Ha DR, Jenkins TC, Kaye KS, Lewis JS 2nd, Ortwine JK, Pogue JM, Spivak ES, Stevens MP, Vaezi L, Tamma PD (2021). Optimizing the management of uncomplicated gram-negative bloodstream infections: consensus guidance using a modified delphi process. Open Forum Infect Dis.

[30] Howard-Anderson J, Dai W, Yahav D, Hamasaki T, Turjeman A, Koppel F, Franceschini E, Hill C, Sund Z, Chambers HF, Fowler VG Jr, Boucher HW, Evans SR, Paul M, Holland TL, Doernberg SB (2022). A desirability of outcome ranking analysis of a randomized clinical trial comparing seven versus fourteen days of antibiotics for uncomplicated gram-negative bloodstream infection. Open Forum Infect Dis.

